# Serum Collagen Type II Cleavage Epitope and Serum Hyaluronic Acid as Biomarkers for Treatment Monitoring of Dogs with Hip Osteoarthritis

**DOI:** 10.1371/journal.pone.0149472

**Published:** 2016-02-17

**Authors:** José M. Vilar, Mónica Rubio, Giuseppe Spinella, Belén Cuervo, Joaquín Sopena, Ramón Cugat, Montserrat Garcia-Balletbó, Juan M. Dominguez, Maria Granados, Asta Tvarijonaviciute, José J. Ceron, José M. Carrillo

**Affiliations:** 1 Departamento de Patología Animal, Universidad de las Palmas de Gran Canaria, Arucas, Las Palmas, Spain; 2 Departamento Medicina y Cirugía Animal, Cátedra García Cugat, Universidad CEU Cardenal Herrera, Valencia, Spain; 3 Department of Veterinary Medical Sciences, University of Bologna, Ozzano dell’Emilia, Bologna, Italy; 4 Artroscopia GC, Hospital Quirón, Barcelona, Spain; 5 Departamento de Medicina y Cirugía Animal, Universidad de Córdoba, Córdoba, Spain; 6 Departamento de Medicina y cirugía animal, Universidad de Murcia, Murcia, Spain; University of Massachusetts Medical, UNITED STATES

## Abstract

The aim of this study was to evaluate the use of serum type II collagen cleavage epitope and serum hyaluronic acid as biomarkers for treatment monitoring in osteoarthritic dogs. For this purpose, a treatment model based on mesenchymal stem cells derived from adipose tissue combined with plasma rich in growth factors was used. This clinical study included 10 dogs with hip osteoarthritis. Both analytes were measured in serum at baseline, just before applying the treatment, and 1, 3, and 6 months after treatment. These results were compared with those obtained from force plate analysis using the same animals during the same study period. Levels of type II collagen cleavage epitope decreased and those of hyaluronic acid increased with clinical improvement objectively verified via force plate analysis, suggesting these two biomarkers could be effective as indicators of clinical development of joint disease in dogs.

## Introduction

Osteoarthritis (OA) affects the articular cartilage in both human [1] and veterinary patients [2], making its treatment challenging for investigators and clinicians.

Alterations in articular cartilage depend on the balance between the synthesis and degradation of the cartilage matrix. These variations could be monitored by measuring the molecules (biological markers) derived from this synthesis and degradation, which are released in biological fluids [3]. In this sense, synovial, serum and/or urinary assays of bone and cartilage markers are becoming less invasive alternatives to osteochondral biopsy for assessing the response of the articular components to disease and injury [4]. Biomarkers could be useful not only for the diagnosis and early detection of disease, but also for treatment monitoring, because radiographic diagnosis is not entirely conclusive due to its low sensitivity to detect small changes in the initial stages of pathology [5]. At this time, a biomarker, or combination of biomarkers, as a tool for the evaluation of OA has not been fully established, but because it is a simple process that is associated with low cost, easy collection, and short examination time relative to other methods, studies are contributing to establish a full methodology for the diagnosis and monitoring of this disease based on biomarkers [6].

Most of the biomarkers used in joint disease are articular cartilage components such as chondroitin sulphate (CS), keratan sulphate (KS), hyaluronic acid (HA), or type II collagen, among others. Because they are articular cartilage components, some of these biomarkers, alone or together, could have the potential to provide clinically useful indices of the effects of isolated joint injury, the progression of joint changes, and/or the response to therapy [7].

Type II collagen is the major structural protein of cartilage and accounts for approximately 50% of the extracellular cartilage matrix. Fragments derived from collagen degradation have been investigated as potential markers for remodeling cartilage pathologies such as OA [8]. Among the wide variety of type II collagen degradation products, a neoepitope in type II collagen that is generated by the intrahelical cleavage of collagenases (C2C) has been well studied in vivo [9]. However, few studies have been developed about C2C concentration as a diagnostic tool in OA dogs, in which synovial fluid [10,11] and serum [4,12] levels of C2C seem to be significantly increased.

Another major component of synovial fluid and the extracellular matrix, HA is a high molecular weight glycosaminoglycan synthesized by chondrocytes and synovial fibroblasts. Some studies [6,13] have shown that HA levels in serum are increased in human patients with OA, and that this increase is considered a reliable biomarker reflective of cartilage damage and synovitis in these patients. However, in the dog, conflicting results have been reported: some authors showed similar results as in humans [14,15], and others reported that HA levels in dogs seem to decrease [16] or remain invariable [17] when OA is present. Furthermore, recent studies relied on a clinically subjective scoring system to demonstrate an increase in HA serum levels coinciding with improved clinical condition in OA dogs [18,19].

In human patients, a direct correlation between C2C, HA levels, and OA pain has been demonstrated [13]; however, in the literature there are no studies correlating serum levels of biomarkers such as HA or C2C with objective evolution of symptoms of OA in dogs, mainly because in domestic animals, quantification of pain is difficult to achieve.

In the past 5 years, the use of mesenchymal stem cell-based (MSC) therapies and plasma rich in growth factors (PRGF) for repair and regeneration in OA has become a new avenue of treatment as an alternative to surgery [20, 21]. The hypothesis of this study was that serum C2C and HA could serve as objective biomarkers in order to evaluate the effectiveness of OA treatment in dogs. For this reason, the aim of this work was to serially measure these biomarkers at different times in OA dogs treated with a combination of MSC and PRGF, and evaluate these measurements in relation to functional status measured by a kinetic force platform.

## Materials and Methods

### Animals

Ten client-owned, presa canario dogs with moderate to severe OA associated to hip dysplasia were enrolled for inclusion in this study. The body weight of enrolled dogs ranged from 47 to 58.3 kg, and ages were 3 to 7 years. Radiographs confirmed the presence of bilateral OA compatible with D and E degrees of hip dysplasia as defined by the Fédération Cynologique Internationale [World Canine Organization]. Dogs with D degree dysplasia showed obvious deviation from the norm, with evidence of a shallow acetabulum, flattened femoral head, poor joint congruency, and in some cases, subluxation with marked changes of the femoral head and neck. The E-degree dysplasic dogs showed complete dislocation of the hip and severe flattening of the acetabulum and femoral head [22]. Additional radiographs of knee and elbow joints were taken in order to ensure that hip OA was the unique reason for the observed clinical signs. A complete clinical evaluation (physical examination, including vital signs and neurologic and orthopedic exams) assured that general health was otherwise normal. In addition, basic hematology and blood biochemistry parameters were obtained prior to and after the study in order to determine if treatment had non-desirable systemic effects.

The research protocol was revised and authorized by the Ethical Committee of Animal Welfare (CEBA) of the University of Las Palmas de Gran Canaria. The owners of each animal gave permission and signed a written consent form.

### Adipose tissue biopsy and culture of MSCs

A 120-mL blood sample was obtained under aseptic conditions and processed with the DogStem kit (Vet-stem, Biopharma, Belgium), and then, under general anesthesia with 3% sevoflurane, a biopsy of 20 g subcutaneous fat tissue from the inguinal region was obtained through a surgical incision. Immediately, the fat biopsy and blood were sent at 4°C for cell isolation and amplification to the Fat-Stem Laboratory (Belgium). The adipose tissue was processed according to standardized Fat-Stem Laboratory procedures and in accordance with good manufacturing practices (GMP) laboratory regulations. Briefly, the derived adipose tissue was digested enzymatically, washed and centrifuged several times to obtain a concentrate of cells. Subsequently, the mixture of cells was grown in a bioreactor environment with controlled temperature, oxygen, and CO_2_ control. After cultivation in several media, the following parameters were assessed in the cells in order to evaluate the quality of the treatment: fibroblastic morphology (spindle shaped); adherence to glass or plastic when cultured; ease of trypsinization; color of the cultivation media; proliferation time (doubling time) ± 75 hours; presence and absence of surface antigens that determine cellular identity; capacity to be induced in vitro to differentiate into osteoblasts, chondrocytes, and adipocytes; and viability. These parameters coincide with the three minimal criteria standards for defining MSCs suggested by the International Society for Cellular Therapy: adherence to plastic, specific surface antigen expression, and multipotent differentiation potential [23]. Finally, the cells were expanded, and 2 weeks after biopsy, 30 million cells were sent for clinical evaluation in two 2-mL tubes containing 15 million MSCs per tube [24].

Finally, PRGF was prepared following a previously published method [25]: 20 mL blood was aseptically collected in four 4.5-mL citrate tubes, and then centrifuged for 8 minutes at 460 × *g*. Before infiltration, the PRGF was activated with 5% of its volume with 10% calcium chloride. Then, PRGF was associated to MSCs, and the resulting 4-mL solution was injected aseptically into both hip joints through conventional hip arthrocentesis sites under sedation with dexmedetomidine (dexdomitor, Zoetis, Madrid, Spain). After the injection, the dogs were sent home and the owners given instructions on the administration of cephalosporin (20 mg/kg/12h, PO) and omeprazole (0.7 mg/kg/24h, PO) as a prophylactic antibiotic and gastric mucosa protector, respectively, for 7 days.

### Force platform gait analysis

Gait analysis was performed using a single platform mounted in the center of, and level with, a 7-m runway covered by a rubber mat. The mat weight was accounted for by setting to “0 force” with the tare button after the platform was covered. All dogs were leash guided at walk over the force platform by the same handler. Walk velocity was measured by use of a motion sensor (Pasco, CA, USA) positioned 1 m from the platform.

Five valid trials, at a sampling frequency of 250 Hz, were obtained for each dog. A trial was considered valid when the limb fully contacted the force platform, and with the dog walking next to the handler without pulling on the leash. The trial was discarded if the dog was distracted during the measurement, if the limb struck the edge of the force plate, or if any portion of the contralateral paw hit the force plate. A member of the research team evaluated the trial to confirm which limb touched the center of the force platform.

The platform was interfaced with a dedicated computer using DataStudio software (Pasco, CA, USA), which is specially designed for the acquisition, numerical conversion, and storage of data. A team member recorded data from both affected limbs at day 0, 30, 90, and 180 post-treatment; day 0 data also served to determine which limb was more lame in order to use it as reference of lameness evolution. The obtained peak vertical force (PVF) was expressed in Newtons (N) and normalized relative to body weight (%BW) to characterize possible improvement of lameness during treatment with MSCs.

### Collagen and HA serum levels

Blood samples were taken by direct jugular venipuncture from all participants in the early morning immediately before injection (D0), and on days 30, 90, and 180 post-injection. Sera were obtained after blood centrifugation for 15 minutes at 3,000 rpm to measure canine C2C and HA. These two biomarkers were measured by commercial ELISA kits (MyBioSource, San Diego, CA, USA, and Teco Medical, Sissach, Switzerland, respectively). Both assays showed intra and inter assay precision lower than 15% and coefficients of regression higher than 0.98 after linear dilution.

### Statistical analysis

Parameters were estimated by using the free R statistical software (https://www.r-project.org/). Data were assessed for normality with the Shapiro-Wilk test, and the nonparametric Kruskal-Wallis and Mann-Whitney U tests were used to compare non-categorical variables at each follow-up time. A repeated measure ANOVA with a post-hoc Tukey test or related-samples Wilcoxon signed rank test was used to evaluate differences between times, as necessary. For correlation between biomarkers and PVF, Pearson correlation coefficient was used.

## Results

The animals had a mean body weight of 52.8 ± 3.4 kg and a mean age of 4.7± 1.2 years.

### PVF

The mean value for walking velocity of dogs was 1.6 ± 0.5 m/sec. No significant difference in walking velocity existed between dogs (P = 0.063).

**Fig 1:**
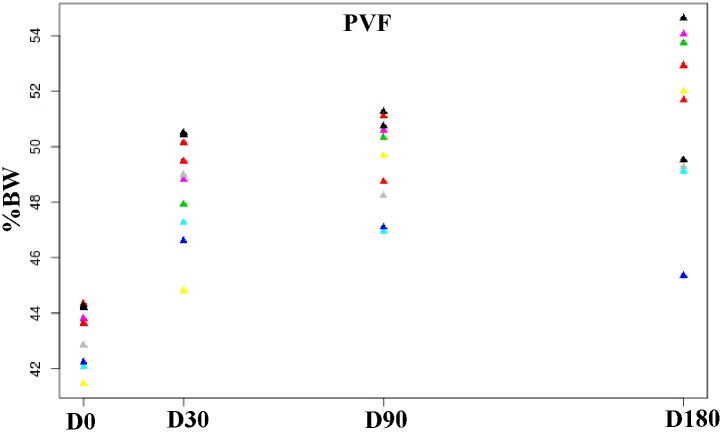
Scatterplot showing evolution of PVF after treatment at the 180-day follow-up period. Each color corresponds to one dog. The graph shows how PVF increases in terms of percent body weight (%BW). The Y axis minimum was set at 40% BW. Unit = Newton.

Mean values of PVF are summarized in Table 1. Results showed that differences in PVF between D0 and the other periods were all significant (P < 0.001). D30 and D180 also showed significant differences (P < 0.001). Fig 1 shows how PVF values increased progressively, with higher improvement within the first 30 days(Fig 1). At D180, PVF values were similar to those previously reported for sound dogs of the same breed (about 48% BW) [20].

**Table 1:** PVF expressed as mean ± SD of %BW.

	**D0**	**D30**	**D90**	**D180**
**D0**	43.27 ± 1.04	P ˂ 0.001	P ˂ 0.001	P ˂ 0.001
**D30**		48.5 ± 1.85	P = 0.19	P ˂ 0.001
**D90**			49.47 ± 1.62	P = 0.77
**D180**				51.23 ± 2.89

D = day. P < 0.05 is considered statistically significant. Unit = Newton.

### Biomarkers

#### C2C

**Fig 2:**
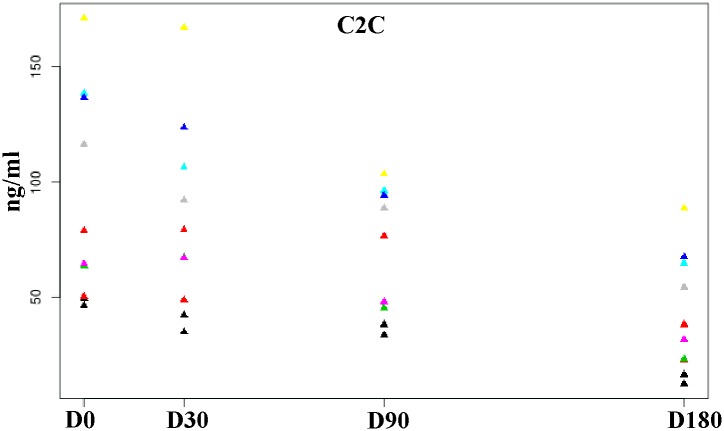
Scatterplot showing decrease of C2C serum levels (ng/ml) after treatment at the 180-day follow-up period. Each color corresponds to one dog.

Mean values of serum C2C are summarized in Table 2. Results showed that differences between D0 and D180 and between D30 and D180 were significant (P = 0.041 and 0.014, respectively). The graph in Fig 2 shows how serum levels decreased(Fig 2).

**Table 2:** Serum levels (ng/ml) of C2C expressed as mean ± SD.

	**D0**	**D30**	**D90**	**D180**
**D0**	91.51 ± 45.25	P = 0.724	P = 0.169	P = 0.041
**D30**		82.94 ± 40.88	P = 0.353	P = 0.014
**D90**			66.14 ± 28.08	P = 0.158
**D180**				41.91 ± 25.51

P < 0.05 is considered statistically significant.

#### HA

**Fig 3:**
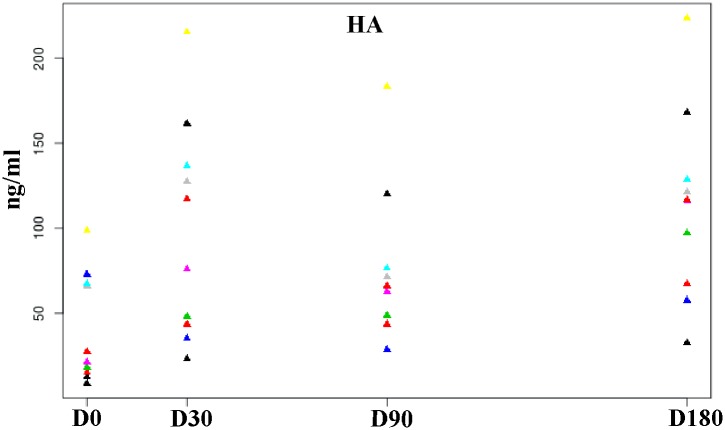
Scatterplot showing increase of HA serum levels (ng/ml) after treatment at the 180-day follow-up period. Each color corresponds to one dog.

Mean values of serum HA are summarized in Table 3. Differences between D0 and all other periods were all significant (P < 0.049). Differences between D90 and D180 were also significant (P = 0.022). The graph in Fig 3 shows how serum levels increased (Fig 3).

**Table 3:** Serum levels (ng/ml) of HA expressed as mean ± SD.

	**D0**	**D30**	**D90**	**D180**
**D0**	40.87 ± 32.1	P = 0.030	P = 0.049	P = 0.011
**D30**		98.40 ± 63.23	P = 0.066	P = 0.49
**D90**			74.45 ± 45.74	P = 0.022
**D180**				112.88 ± 55.21

P < 0.05 is considered statistically significant.

### Correlation

**Fig 4:**
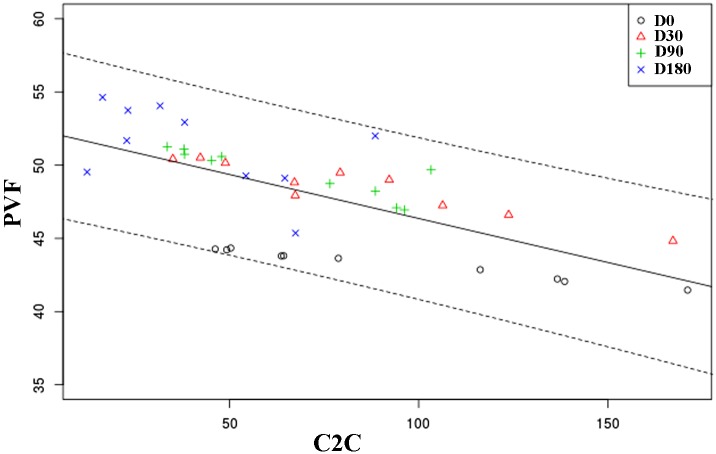
Scatterplot showing correlation between C2C and PVF at the 180-day follow-up period. Each symbol represents the values of any individual dog at the same checking period. The solid line represents the trend line. Dotted lines represent the 95% confidence interval. The Y axis minimum was set at 35% BW.

**Fig 5:**
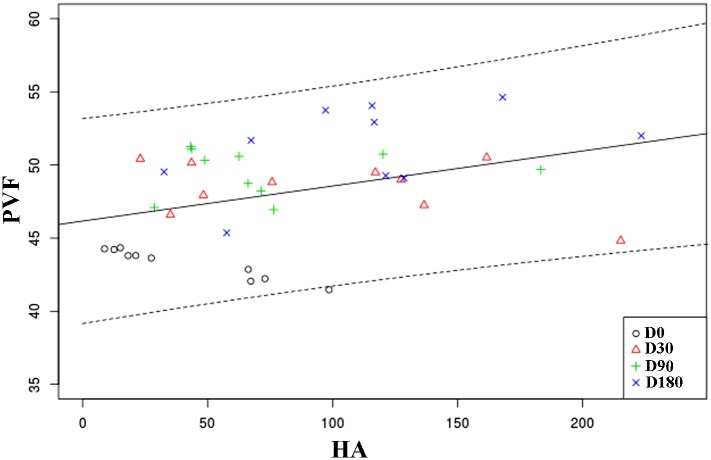
Scatterplot showing correlation between HA and PVF at the 180-day follow-up period. Each symbol represents the values of any individual dog at the same checking period. The solid line represents the trend line. Dotted lines represent the 95% confidence interval. The Y axis minimum was set at 35% BW.

A significant inverse correlation (Pearson r = -071) was found between C2C and PVF (P = 0.008) (Fig 4). However, a positive association between HA and PVF (Pearson r = 0.477) could be demonstrated (P = 0.011) (Fig 5).

## Discussion

In this study, the correlation between serum levels of C2C and HA biomarkers and objective evolution of PVF values in dogs with hip OA before and after treatment has been demonstrated.

The overproduction of destructive substances and inflammation mediators in OA give rise to a balance in favor of articular cartilage catabolism [26]. The collagen framework becomes disrupted, and the proteoglycans content of the articular cartilage diminishes. Proteoglycan fragments and the breakdown products of type II collagen, are liberated in increased concentrations to reach the synovial fluid and the serum [17,27]. All of these variations could be monitored by measuring the molecules derived from this synthesis and degradation in serum [3].

The diagnosis of OA is generally based on clinical and radiographic changes that occur in the later stages of the disease; this technique shows the bone texture at a given moment. Radiology has been widely used for its ease of availability and accessibility, although there is controversy as to its utility as a diagnostic method for evaluating responses to different treatments [28]. For this reason, biomarkers could aid in monitoring disease status, predicting disease progression, and studying the effects of novel therapeutic interventions in a variety of joint diseases [7].

There is controversy in the usefulness of C2C as a biomarker in OA; while some authors determined an increase of C2C serum concentrations in human and dog patients suffering from injured joints [4,10,12,29], other authors suggested that collagenase-specific degradation of type II collagen in articular cartilage may not be involved, at least in early stages of naturally occurring OA [30]. In our study, the results show that after one intraarticular injection of MSC + PRGF, a concurrent decrease in C2C level and OA improvement occurred, as objectively measured by force plate analysis.

Although previous studies have suggested that HA may be used as a biomarker in OA dogs [17,18], some controversy exists between those reports of elevation in HA in relation to the degree of OA in humans [6,13] and dogs [14,15], and those performed in dogs in which the opposite seems to occur: levels of HA in OA animals were lower than those in healthy animals and seemed to increase when animals improved with physical or surgical therapy [16,18,19]. Our results are consistent with these findings in that the treated dogs’ HA serum levels were significantly higher compared with D0 at 1, 3, and 6 months follow-up after the MSC + PRGF intraarticular injection. However, although our results showed statistical correlation with PVF values, we observed a high variability. These results could have several possible explanations: the first one could be the diurnal-related changes in this marker that have been previously described [31]; to account for such changes, comparisons should be performed only when HA levels are measured in the same hour. Secondly, food quantity and characteristics seem to significantly affect HA levels [32]; therefore, comparisons should be made only in patients submitted to the same feeding protocol and the same period of fast. Third, HA levels in OA could behave differently when different species or even different breeds are compared. In addition, some pathologies, especially those with hepatic damage, have been demonstrated to alter serum HA levels [15,33]; therefore, HA studies should include animals with normal hematological and blood biochemical profiles.

In summary, HA serum levels in dogs could not be modified only by structural or functional changes in joints, although these levels showed a significant elevation when condition improved; further research should clarify the role of HA as an indicator of functional status in dogs to determine if HA behaves in the same manner in dogs as in humans [13]. This aim could be achieved by using a larger study group with the same research protocol (breed, food, time of blood samples, etc.).

Based in our results, the C2C and HA biomarkers might be a promising way to objectively measure improvement after OA treatment in canine joints. An association was seen between decreased C2C and increased HA serum levels and improvement in lameness.

## Conclusion

Based on the results from the current study, C2C and HA serum concentration are adequate and objective biomarkers for testing the limb functionality of treated OA joints in dogs.

## Supporting Information

S1 ARRIVE Checklist ARRIVE guidelines. (PDF)

## References

[1] Juni P, Reichenbach S, Dieppe P. Osteoarthritis: rational approach to treating the individual. Best Pract Res Clin Rheumatol. 2006; 20: 721–740. 16979535 10.1016/j.berh.2006.05.002

[2] Malek S, Sample SJ, Schwartz Z, Nemke B, Jacobson PB, Cozzi EM, et al Effect of analgesic therapy on clinical outcome measures in a randomized controlled trial using client-owned dogs with hip osteoarthritis. BMC Vet Res. 2012; 8: 185 10.1186/1746-6148-8-185 23035739 PMC3527270

[3] Ramonda R, Lorenzin M, Modesti V, Campana C, Ortolan A, Frallonardo P, et al Serological markers of erosive hand osteoarthritis. Eur J Intern Med. 2013; 24: 11–15. 10.1016/j.ejim.2012.10.002 23102569

[4] Goranov NV. Serum markers of lipid peroxidation, antioxidant enzymatic defense, and collagen degradation in an experimental (Pond-Nuki) canine model of osteoarthritis. Vet Clin Pathol. 2007; 36: 192–195. 17523095 10.1111/j.1939-165x.2007.tb00208.x

[5] Bedson J, Croft P. The discordance between clinical and radiographic knee osteoarthritis: a systematic search and summary of the literature. BMC Musculoskelet Disord. 2008; 9: 116 10.1186/1471-2474-9-116 18764949 PMC2542996

[6] Sasaki E, Tsuda E, Yamamoto Y, Iwasaki K, Inoue R, Takahashi I, et al Serum hyaluronan levels increase with the total number of osteoarthritic joints and are strongly associated with the presence of knee and finger osteoarthritis. Int Orthop. 2013; 37: 925–930. 10.1007/s00264-013-1849-x 23508866 PMC3631499

[7] Lotz M, Martel-Pelletier J, Christiansen C, Brandi M, Bruyère O, Chapurlat R, et al Value of biomarkers in osteoarthritis: current status and perspectives. Ann Rheum Dis. 2013; 72:1756–1763. 10.1136/annrheumdis-2013-203726 23897772 PMC3812859

[8] Garnero P, Delmas P. Biomarkers in osteoarthritis. Curr Opin Rheumatol. 2003; 15: 641–646. 12960494 10.1097/00002281-200309000-00020

[9] Poole AR, Ionescu M, Fitzcharles MA, Billinghurst RC. The assessment of cartilage degradation in vivo: development of an immunoassay for the measurement in body fluids of type II collagen cleaved by collagenases. J Immunol Methods. 2004; 294: 145–153. 15637808 10.1016/j.jim.2004.09.005

[10] Chu Q, Lopez M, Hayashi K, Ionescu M, Billinghurst RC, Johnson KA, et al Elevation of a collagenase generated type II collagen neoepitope and proteoglycan epitopes in synovial fluid following induction of joint instability in the dog. Osteoarthritis Cartilage. 2002; 10: 662–669. 12479389 10.1053/joca.2002.0812 PMC2048684

[11] Prink A, Hayashi K, Kim SY, Kim J, Kapatkin A. Evaluation of a collagenase generated osteoarthritis biomarker in the synovial fluid from elbow joints of dogs with medial coronoid disease and unaffected dogs. Vet Surg. 2010; 39: 65–70. 10.1111/j.1532-950X.2009.00604.x 20210947

[12] Matyas JR, Atley L, Ionescu M, Eyre DR, Poole AR. Analysis of cartilage biomarkers in the early phases of canine experimental osteoarthritis. Arthritis Rheum. 2004; 50: 543–552. 14872497 10.1002/art.20027

[13] Ishijima M, Watari T, Naito K, Kaneko H, Futami I, Yoshimura-Ishida K, et al Relationships between biomarkers of cartilage, bone, synovial metabolism and knee pain provide insights into the origins of pain in early knee osteoarthritis. Arthritis Res Ther. 2011; 13: 22. 10.1186/ar3246 PMC3241366 21320321

[14] Thonar EJ, Masuda K, Lenz ME, Hauselmann HJ, Kuettner KE, Manicourt DH. Serum markers of systemic disease processes in osteoarthritis. J Rheumatol Suppl. 1995; 43: 68–70. 7752142

[15] Arican M, Carter SD, May C, Bennett D. Hyaluronan in canine arthropathies. J Comp Pathol. 1994; 111: 185–195. 7806704 10.1016/s0021-9975(05)80050-7

[16] Nganvongpanit K, Itthiarbha A, Ong-Chai S, Kongtawelert P. Evaluation of serum chondroitin sulfate and hyaluronan: biomarkers for osteoarthritis in canine hip dysplasia. J Vet Sci. 2008; 9: 317–325. 18716453 10.4142/jvs.2008.9.3.317 PMC2811845

[17] Leipold HR, Goldberg RL, Lust G. Canine serum keratan sulfate and hyaluronate concentrations. Relationship to age and osteoarthritis. Arthritis Rheum. 1989; 32: 312–321. 2522784 10.1002/anr.1780320313

[18] Budsberg SC, Lenz ME, Thonar EJ. Serum and synovial fluid concentrations of keratan sulfate and hyaluronan in dogs with induced stifle joint osteoarthritis following cranial cruciate ligament transection. Am J Vet Res. 2006; 67: 429–432. 16506904 10.2460/ajvr.67.3.429

[19] Nganvongpanit K, Tanvisut S, Yano T, Kongtawelert P. Effect of Swimming on Clinical Functional Parameters and Serum Biomarkers in Healthy and Osteoarthritic Dogs. ISRN Veterinary Science 2014 1 9 459809. 10.1155/2014/459809 PMC4060742 24977044

[20] Vilar JM, Batista M, Morales M, Santana A, Cuervo B, Rubio M, et al Assessment of the effect of intraarticular injection of autologous adipose-derived mesenchymal stem cells in osteoarthritic dogs using a double blinded force platform analysis. BMC Vet Res. 2014; 10: 143 10.1186/1746-6148-10-143 24984756 PMC4085658

[21] Vilar JM, Morales M, Santana A, Spinella G, Rubio M, Cuervo B, et al Controlled, blinded force platform analysis of the effect of intraarticular injection of autologous adipose-derived mesenchymal stem cells associated to PRGF-Endoret in osteoarthritic dogs. BMC Vet Res. 2013; 9: 131 10.1186/1746-6148-9-131 23819757 PMC3716942

[22] Piermattei D, Flo G, DeCamp C. Hip joint In: Fathman L, editor. Brinker, Piermattei and Flo’s handbook of small orthopedics and fracture repair. Saunders; 2006 pp. 461–511.

[23] Dominici M, Le Blanc K, Mueller I, Slaper-Cortenbach I, Marini F, Krause D, et al Minimal criteria for defining multipotent mesenchymal stromal cells. The International Society for Cellular Therapy position statement. Cytotherapy. 2006; 8: 315–317. 16923606 10.1080/14653240600855905

[24] Cuervo B, Rubio M, Sopena J, Dominguez JM, Vilar J, Morales M, et al Hip osteoarthritis in dogs: a randomized study using mesenchymal stem cells from adipose tissue and plasma rich in growth factors. Int J Mol Sci. 2014; 15: 13437–13460. 10.3390/ijms150813437 25089877 PMC4159804

[25] Anitua E, Sánchez M, Orive G, Andía I. The potential impact of the preparation rich in growth factors (PRGF) in different medical fields. Biomaterials. 2007; 28: 4551–6450. 17659771 10.1016/j.biomaterials.2007.06.037

[26] Kapoor M, Martel-Pelletier J, Lajeunesse D, Pelletier JP, Fahmi H. Role of proinflammatory cytokines in the pathophysiology of osteoarthritis. Nat Rev Rheumatol. 2011; 7: 33–42. 10.1038/nrrheum.2010.196 21119608

[27] Hegemann N, Kohn B, Brunnberg L, Schmidt MF. Biomarkers of joint tissue metabolism in canine osteoarthritic and arthritic joint disorders. Osteoarthritis Cartilage. 2002;. 10: 714–721. 12202124 10.1053/joca.2002.0820

[28] Hirvasniemi J, Thevenot J, Immonen V, Liikavainio T, Pulkkinen P, Jämsä T, et al Quantification of differences in bone texture from plain radiographs in knees with and without osteoarthritis. Osteoarthritis Cartilage. 2014;. 22: 1724–1731. 10.1016/j.joca.2014.06.021 25278081 PMC4587537

[29] Kumahashi N, Swärd P, Larsson S, Lohmander LS, Frobell R, Struglics A. Type II collagen C2C epitope in human synovial fluid and serum after knee injury—associations with molecular and structural markers of injury. Osteoarthritis Cartilage. 2015 4 29 pii: S1063-4584(15)01142-5. 10.1016/j.joca.2015.04.022 25937025

[30] Hayashi K, Kim SY, Lansdowne JL, Kapatkin A, Déjardin LM. Evaluation of a collagenase generated osteoarthritis biomarker in naturally occurring canine cruciate disease. Vet Surg. 2009; 38: 117–121. 10.1111/j.1532-950X.2008.00446.x 19152626

[31] Gordon CD, Stabler TV, Kraus VB. Variation in osteoarthritis biomarkers from activity not food consumption. Clin Chim Acta. 2008 12 10.1016/j.cca.2008.07.031 PMC2586038 18727924

[32] Garnero P, Gourley I, Mareau E, Durn B, Hickey L, Cohen S. Biological variability of biochemical markers of bone, cartilage, and synovial metabolism in patients with knee osteoarthritis: effect of food intake. Osteoarthritis Cartilage. 2007; 15: 68.

[33] Seki M, Asano K, Sakai M, Kanno N, Teshima K, Edamura K, et al Serum hyaluronic acid in dogs with congenital portosystemic shunts. J Small Anim Pract. 2010; 51: 260–263. 10.1111/j.1748-5827.2010.00934.x 20402843

